# Advancements in prokaryotic systematics and the role of Bergey's International Society for Microbial Systematicsin addressing challenges in the meta-data era

**DOI:** 10.1093/nsr/nwae168

**Published:** 2024-05-13

**Authors:** Jian-Yu Jiao, Rashidin Abdugheni, Dao-Feng Zhang, Iftikhar Ahmed, Mukhtiar Ali, Maria Chuvochina, Svetlana N Dedysh, Xiuzhu Dong, Markus Göker, Brian P Hedlund, Philip Hugenholtz, Kamlesh Jangid, Shuang-Jiang Liu, Edward R B Moore, Manik Prabhu Narsing Rao, Aharon Oren, Ramon Rossello-Mora, Bhagwan Narayan Rekadwad, Nimaichand Salam, Wensheng Shu, Iain C Sutcliffe, Wee Fei Aaron Teo, Martha E Trujillo, Stephanus N Venter, William B Whitman, Guoping Zhao, Wen-Jun Li

**Affiliations:** State Key Laboratory of Biocontrol, Guangdong Provincial Key Laboratory of Plant Resources and Southern Marine Science and Engineering Guangdong Laboratory (Zhuhai), School of Life Sciences, Sun Yat-Sen University, Guangzhou 510275, China; State Key Laboratory of Desert and Oasis Ecology, Key Laboratory of Ecological Safety and Sustainable Development in Arid Lands, Xinjiang Institute of Ecology and Geography, Chinese Academy of Sciences, Urumqi 830011, China; Jiangsu Province Engineering Research Center for Marine Bio-resources Sustainable Utilization & College of Oceanography, Hohai University, Nanjing 210024, China; National Culture Collection of Pakistan (NCCP), Land Resources Research Institute (LRRI), National Agricultural Research Centre (NARC), Islamabad 45500, Pakistan; State Key Laboratory of Biocontrol, Guangdong Provincial Key Laboratory of Plant Resources and Southern Marine Science and Engineering Guangdong Laboratory (Zhuhai), School of Life Sciences, Sun Yat-Sen University, Guangzhou 510275, China; The University of Queensland, School of Chemistry and Molecular Biosciences, Australian Centre for Ecogenomics, Queensland 4072, Australia; Winogradsky Institute of Microbiology, Research Center of Biotechnology, Russian Academy of Sciences, Moscow 117312, Russia; State Key Laboratory of Microbial Resources, Institute of Microbiology, Chinese Academy of Sciences, Beijing 100101, China; Leibniz Institute DSMZ – German Collection of Microorganisms and Cell Cultures, Braunschweig D-38124, Germany; School of Life Sciences, University of Nevada, Las Vegas, NV 89154, USA; Nevada Institute of Personalized Medicine, University of Nevada, Las Vegas, NV 89154, USA; The University of Queensland, School of Chemistry and Molecular Biosciences, Australian Centre for Ecogenomics, Queensland 4072, Australia; Bioenergy Group, MACS Collection of Microorganisms, Agharkar Research Institute, Pune 411004, India; State Key Laboratory of Microbial Resources, Institute of Microbiology, Chinese Academy of Sciences, Beijing 100101, China; State Key Laboratory of Microbial Technology, Shandong University, Qingdao 266237, China; Department of Infectious Disease, Institute for Biomedicine, and Culture Collection University of Gothenburg (CCUG), Institute for Biomedicine, Sahlgrenska Academy, University of Gothenburg, Gothenburg SE-40234, Sweden; Instituto de Ciencias Aplicadas, Facultad de Ingeniería, Universidad Autónoma de Chile, Talca 3460000, Chile; The Alexander Silberman Institute of Life Sciences, The Edmond J. Safra Campus, The Hebrew University of Jerusalem, Jerusalem 9190401, Israel; Marine Microbiology Group, Department of Animal and Microbial Biodiversity, Mediterranean Institute for Advanced Studies (IMEDEA, CSIC-UIB), Esporles 070190, Spain; MicrobeAI Lab, Division of Microbiology and Biotechnology, Yenepoya Research Centre, Yenepoya (Deemed to be University), Mangalore 575018, India; National Agri-Food Biotechnology Institute, Knowledge City, Mohali 140306, India; Institute of Ecological Science, School of Life Science, South China Normal University, Guangzhou 510631, China; Department of Applied Sciences, Faculty of Health and Life Sciences, Northumbria University, Newcastle upon Tyne NE1 8ST, UK; Institute of Biological Sciences, Faculty of Science, Universiti Malaya, Kuala Lumpur 50603, Malaysia; Microbiology and Genetics Department, University of Salamanca, Salamanca 37008, Spain; Department of Biochemistry, Genetics and Microbiology, and Forestry and Agricultural Biotechnology Institute (FABI), University of Pretoria, Pretoria 0028, South Africa; Department of Microbiology, University of Georgia, Athens, GA 30602, USA; Institute of Synthetic Biology, Shenzhen Institutes of Advanced Technology, Chinese Academy of Sciences, Shenzhen 518055, China; State Key Laboratory of Biocontrol, Guangdong Provincial Key Laboratory of Plant Resources and Southern Marine Science and Engineering Guangdong Laboratory (Zhuhai), School of Life Sciences, Sun Yat-Sen University, Guangzhou 510275, China; State Key Laboratory of Desert and Oasis Ecology, Key Laboratory of Ecological Safety and Sustainable Development in Arid Lands, Xinjiang Institute of Ecology and Geography, Chinese Academy of Sciences, Urumqi 830011, China

**Keywords:** prokaryotic systematics, *Bergey's Manual of Systematics of Archaea and Bacteria* (*BMSAB*), Bergey's International Society for Microbial Systematics (BISMiS), meta-data era

## Abstract

Prokaryotes are ubiquitous in the biosphere, important for human health and drive diverse biological and environmental processes. Systematics of prokaryotes, whose origins can be traced to the discovery of microorganisms in the 17th century, has transitioned from a phenotype-based classification to a more comprehensive polyphasic taxonomy and eventually to the current genome-based taxonomic approach. This transition aligns with a foundational shift from studies focused on phenotypic traits that have limited comparative value to those using genome sequences. In this context, *Bergey's Manual of Systematics of Archaea and Bacteria* (BMSAB) and Bergey's International Society for Microbial Systematics (BISMiS) play a pivotal role in guiding prokaryotic systematics. This review focuses on the historical development of prokaryotic systematics with a focus on the roles of BMSAB and BISMiS. We also explore significant contributions and achievements by microbiologists, highlight the latest progress in the field and anticipate challenges and opportunities within prokaryotic systematics. Additionally, we outline five focal points of BISMiS that are aimed at addressing these challenges. In conclusion, our collaborative effort seeks to enhance ongoing advancements in prokaryotic systematics, ensuring its continued relevance and innovative characters in the contemporary landscape of genomics and bioinformatics.

## INTRODUCTION

Prokaryotes, encompassing *Archaea* and *Bacteria*, are crucial, ubiquitous inhabitants of planet Earth. As the most diverse organismal life forms occupying a wide variety of ecological niches, prokaryotes play significant roles in most ecological processes and profoundly impact human health, biotechnology, agriculture and the environment [1–5].

The recognition of the enormous diversity of prokaryotes has progressed slowly over the last century. For instance, the reported number of prokaryotic species was 2703 in 1934 and 6200 in 2004, increasing to 11 482 in 2013 and 17 137 in 2020 [6–9]. Since that time, approximately one thousand new species have been described each year so that the number of species with validly published names under the International Code of Nomenclature of Prokaryotes (ICNP), as of 29 April, 2024, is 24 702 (https://lpsn.dsmz.de/text/numbers). Additionally, more than 2500 *Candidatus* taxa, many of which were proposed using sequence data as type materials, have been assigned to prokaryotes that have not yet been isolated or deposited in culture collections [10]. With the rapid development and widespread application of metagenomic sequencing technologies and bioinformatics, enormous amounts of information have been generated on prokaryotic diversity and function. Nevertheless, lack of systematic studies of these vast amounts of data hampers communication in all the related fields of microbiology [11].

A comprehensive and well-ordered taxonomy for prokaryotes serves to improve communication among scientists and facilitate comparative and evolutionary biology. It is essential for harnessing microbial potential for applications in various fields, facilitating the recognition of their similarities and differences, and efficient dissemination of the relevant knowledge. The discipline of prokaryotic systematics aims to infer the evolutionary relationships, define the taxonomic names and positions, and describe features of related prokaryotes [6,7]. This discipline has a rich and intricate history, dating back to the 17th-century discovery of prokaryotes through invention and further enhancement of light microscopy [12]. This period may be considered as the genesis of the phenotype-based taxonomy of microorganisms, which eventually led to the evolution of modern polyphasic taxonomy [13–15]. These developments occurred in parallel with research progress in molecular biology of cells, microbial physiology and biogeochemistry [16].

*Bergey's Manual of Systematics of Archaea and Bacteria* (*BMSAB*), a reference resource and rigorous handbook on prokaryotic systematics, made its debut in 1923 as *Bergey's Manual of Determinative Bacteriology* under the guidance of the editorial chair David Hendricks Bergey (1860–1937) [17]. Evolving over the years, it underwent name changes, transitioning into *Bergey's Manual of Systematic Bacteriology* in 1984, and, in 2015, adopting its current title of *Bergey's Manual of Systematics of Archaea and Bacteria* [18]. This online-only inception of the Manual marked a pivotal milestone in grasping different aspects of the currently available information on prokaryotic taxa and their classification and more timely dissemination of information.

Widely acknowledged as the ‘Bible’ of prokaryotic taxonomy, *BMSAB* aims to offer detailed descriptions of the systematics, ecology, physiology, morphology and other biological properties of all described prokaryotes. The establishment of Bergey's Manual Trust (BMT) in 1936 [19], with its primary responsibility being the periodic review and revision of *BMSAB*, reflects the commitment to maintaining accuracy and relevance. Comprising leading experts in prokaryotic systematics, the BMT integrates discoveries, advancements and principles related to prokaryotic taxonomy into the *BMSAB* chapters [20,21]. Recognizing the imperative for international collaboration in advancing prokaryotic systematics, the BMT took the initiative to support the creation of Bergey's International Society for Microbial Systematics (BISMiS) in 2009. The society welcomed members from the scientific community with a keen interest in prokaryotic systematics. BISMiS members convene biennially, fostering a platform for scholarly exchange and collaboration. The inaugural meeting took place in Beijing, China, in 2011, marking the beginning of a collective effort to propel the field of prokaryotic systematics forward.

As prokaryotic systematics advanced, so did the evolution of codes of nomenclature that are regulating naming of prokaryotes. The historical decision by bacteriologists to separate bacterial nomenclature from the International Code of Botanical Nomenclature (ICBN) in 1948, driven by fundamental differences in taxonomic approaches and the emphasis on live cultures as nomenclature types, gave rise to the International Bacteriological Code of Nomenclature (IBCN) [22]. This regulatory framework aimed to standardize the naming of prokaryotes to improve clarity and stability. After the 1975 revision, it became mandatory that prokaryotic names proposed before 1980 be included in the Approved Lists of Bacterial Names [23], published in 1980, to maintain standing in nomenclature. Thereafter, proposed names of prokaryotes had to be published in the *International Journal of Systematic and Evolutionary Microbiology* (*IJSEM*), formerly the *International Journal of Systematic Bacteriology* (*IJSB*), or, if published outside of *IJSEM*, be included in the IJSEM Validation List, to be validly published.

Over the years, ‘The Code’ of prokaryotic nomenclature has undergone several revisions. In 1999, it was renamed the International Code of Nomenclature of Prokaryotes. The 2008 revision of the ICNP incorporated a requirement to deposit viable cultures of each type strain in two culture collections located in different countries for the valid publication of a name. However, this requirement precluded the validation of names for many ‘unculturable’ prokaryotes and various taxa that are difficult to preserve, store or disseminate. In 2015, a proposal was made to revise the ICNP so that DNA sequence data could serve as an alternative nomenclatural type [24]. This idea was supported by multiple researchers of different disciplines as a solution to the nomenclature of the fastidious and, thus far, unculturable prokaryotes [25]. However, the proposal was rejected by the International Committee on Systematics of Prokaryotes (ICSP) [26]. In response, the Code of Nomenclature of Prokaryotes Described from DNA Sequence Data (or the SeqCode) was completed in 2022, enabling an alternative valid publication of names based on genome sequences, metagenome-assembled genomes (MAGs) or single-cell amplified genomes (SAGs) [27]. This action, while facilitating advancements based on contemporary genomic approaches, has led to the coexistence of two separate codes of nomenclature for prokaryotes that contradict each other. BISMiS is playing a reconciliatory role in order to advance common interests in prokaryotic systematics by facilitating knowledge exchange within the scientific community.

In this review, we thoroughly examine the current methods and advancements in prokaryotic systematics and the contributions made by *BMSAB* and BISMiS in this regard. Our objective is to offer insights into the history, current status and future of microbial systematics, as viewed through the prism of the leadership of BISMiS. We aim to highlight advancements, provide perspectives on the challenges and opportunities, and serve as a valuable reference and source of inspiration for researchers in this field.

## HISTORY AND DEVELOPMENT OF PROKARYOTIC SYSTEMATICS

Prokaryotic systematics is concerned with the classification, identification and determination of evolutionary relationships of microorganisms. Its origins can be traced back to the 17th century when Dutch draper and amateur microscopist Antonie van Leeuwenhoek (1632–1723) made pioneering observations of microscopic organisms, referring to them as ‘animalcules’, and providing detailed descriptions of their shapes and movements [28]. Advancements in microscopy over the centuries allowed for more in-depth studies of microbes in much greater detail. Phenotype-based classification and description of prokaryotes can be dated back to the Linnaean approach to naming life forms, such as Müller's use of *Vibrio* and *Monas* (O.F. Müller 1773, 1786) [17,29,30]. The German mycologist Johann Heinrich Friedrich Link (1767–1851) described the first bacterium, *Polyangium vitellinum*, in 1809, marking the initiation of bacterial species nomenclature [31]. In 1875, microbiologist Ferdinand Cohn attempted to establish formal rules of microbial nomenclature [17]. Bacterial genera such as *Corynebacterium, Mycobacterium* and *Actinomyces* were first reported and described in 1896 by K.B. Lehman and R. Neumann [32].

Methods used to classify and identify prokaryotes have evolved over the years. The early stages of prokaryotic taxonomy focused on morphology and other phenotypic criteria, including cell shape, size and motility [33,34]. Late in the 19th century, Robert Koch and Friedrich Loeffler developed the first pure-culture-based studies of microbes, which fundamentally changed the trajectory of systematics and promoted studies with pure cultures. From these studies, systematic bacteriology dramatically emerged as a distinct field [35]. However, one of the most important breakthroughs in prokaryote taxonomy occurred in the early 1960s just after the discovery of the genetic code, when genome measures such as G + C mol% and DNA-DNA hybridization (DDH) techniques were implemented [36]. DDH especially became the gold standard for numerically circumscribing species, and a species-threshold was established as a DDH similarity of 70%. In 1977, Carl Woese and colleagues introduced rRNA-based phylogenetics into prokaryotic taxonomy, and they classified *Archaea* as a separate group of prokaryotes [37]. While these advancements strengthened prokaryotic taxonomy, challenges persisted, especially at the species ranks, owing to limitations of phylogenetic analyses based on rRNA catalogs or, later, single gene sequences such as those for 5S and 16S rRNAs [38,39].

**Figure 1.**
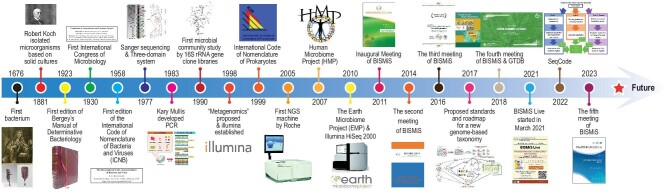
Milestones of prokaryotic systematics [8,13,27,37,57–68].

Modern prokaryotic systematics evolved along with the advancements in biochemistry, analytical chemistry, molecular biology, genetics, DNA sequencing and computing. Early whole-genome approaches such as DDH [40] and determination of G + C contents [41], along with quantitative analysis of cellular components such as lipids, quinones, peptidoglycan and small metabolites [42–46], provided more holistic data sets for comparative biology. Recent decades have seen remarkable progress with the advent of whole-genome sequencing and bioinformatics, which allowed the development of a wide array of quantitative similarity criteria, such as average nucleotide identity (ANI) [47–49], digital DNA-DNA hybridization (dDDH) [50], average amino acid identity (AAI) [51], protein ortholog clusters percentage (POCP) [52] and core genome-based phylogenetics [53–56]. Together, these approaches have improved the resolution and reliability of prokaryotic classifications and assignment at all taxonomic ranks (Fig. 1 and Table 1).

**Table 1.** Significant advancements in prokaryotic systematics.

Year	Name of event	Significance	References
1684	First observation of bacterial cells by Antonie van Leeuwenhoek	Origin of phenotype-based microbial taxonomy	[12]
1773–1786	Introduction of the first bacterial description by O.F. Müller	Introduction of morphological descriptions	[17,29,30]
1809	Nomination of *Polyangium vitellinum* by Johann Heinrich Friedrich Link	Beginning of nomenclature of bacterial species	[31]
1875	Purification of *Bacillus anthracis* by Robert Koch	Beginning of the pure-culture based bacteriology	[58]
1875	Attempt to establish formal rules for microbial nomenclature by Ferdinand Cohn	First attempt at formal microbial nomenclature	[69]
1896	First report and description of bacterial genera by K.B. Lehman and R. Neumann	The first report of bacterial genera	[32]
1901	The first manual of bacterial taxonomy was published by Professor Frederick Dixon Chester	Publication of the first handbook of bacterial taxonomy	[70]
1923	Bergey's *Manual* was published by the American bacteriologist, David Hendricks Bergey and American Society for Microbiology members	The birth of *Bergey's Manual of Determinative Bacteriology*	[8]
1977	Archaea were first classified as a separate group of prokaryotes based on phylogenies derived from 16S rRNA catalogs	The birth of the three-domain system and rRNA-based taxonomy	[13]
1984	Introduction of culture-independent studies of prokaryotic diversity in natural environments based on rRNA	The birth of the culture-independent study of prokaryotic diversity	[5]
1977–2000	Inclusion of DNA-DNA hybridization, GC content analysis	Beginning of the genome-based taxonomy of prokaryotes	[40–43]
2000–2023	Inclusion of dDDH, ANI, core/conserved gene-based phylogenetics, AAI, POCP, MALDI-TOF-MS	Comprehensive phenotype, genotype and protein-based taxonomy of prokaryotes	[47,49–55,67,71–74]

The field has evolved rapidly over the last decade, with advances in DNA sequencing technology and computing making systematics and ecological research increasingly affordable [75]. As early as the late-1990s when 16S rRNA sequencing of genes from environmental samples was introduced [76] it was recognized that most of the prokaryotes had never been cultivated [77]. The sequences of the 5S, 16S and 23S rRNA genes provide little information about the organisms, so it was a major step forward when rapid advancements in single-cell genomics and metagenomics enabled the recovery of genome sequences, SAGs or MAGs, respectively. The rapid discovery of deep lineages of prokaryotes with few or no isolates or co-cultures challenged the existing prokaryotic systematics framework to incorporate organisms that are known only from their genome sequence. The Genome Taxonomy Database (GTDB) was created to address this challenge by providing a phylogenetically consistent and rank-normalized genome-based taxonomy for prokaryotic genomes sourced from the NCBI assembly database [78]. The GTDB, combined with the Genome Taxonomy Database Toolkit (GTDB-Tk) [79], allows SAGs and MAGs to be objectively assigned to taxonomic positions while accounting for different rates of evolution between lineages. Other taxonomic resources such as the Microbial Genomes Atlas (MiGA) [80], also facilitate classifications of genome sequences via comparisons with the known taxa and identification of the most closely related genomic representatives.

## HISTORY AND EVOLUTION OF *BERGEY'S MANUAL OF SYSTEMATICS OF ARCHAEA AND BACTERIA*

*BMSAB* stands as a seminal work in the field of prokaryotic systematics, with a goal to provide a detailed and comprehensive description of prokaryotic taxa. The Manual is in continuous development and provides a compilation of previous efforts acting as a dedicated reference for microbial taxonomy.

The first handbook of bacterial systematics, *A Manual of Determinative Bacteriology*, was published in 1901 by professor Frederick Dixon Chester (1861–1943) [70]. Around 1910–1920, the Committee on Characterization and Classification of Bacterial Types of the Society of American Bacteriologists (SAB, currently American Society for Microbiology) announced the need for a more detailed reference manual for the identification of bacterial species, which promoted the birth of the first edition of Bergey's Manual [59]. In 1923, an editorial committee comprising David Bergey, Francis Harrison, Robert Breed, Bernard Hammer and Frank Huntoon, who were appointed by SAB, published the first edition of the *Bergey's Manual of Determinative Bacteriology* [8]. The birth of ‘Bergey's Manual’ laid a strong foundation for subsequent standardization in microbial taxonomy. Starting in approximately 1920, driven by technological innovations, prokaryotic systematics also underwent rapid theoretical development. The second and third editions of Bergey's Manual were subsequently published in 1925 and 1930, respectively. In 1936, the Bergey's Manual Trust was established to oversee the publication and coordination of various activities related to the Manual [69]. Since the establishment of the Trust, subsequent editors published the fourth through ninth editions of *Bergey's Manual of Determinative Bacteriology* with the final edition released in 1994 [81].

The development in methods used in prokaryotic taxonomy saw a shift towards a more evolutionary framework in the 1970s and 1980s. Between 1984 and 1989, the first edition of *Bergey's Manual of Systematic Bacteriology* was edited by George Garrity and John Holt. This edition relied heavily on rRNA sequences to establish the phylogenetic framework. However, the sequences of many species were not known at that time, so the classification of many species was uncertain. Eleven years later and with an extensive reference rRNA database, the second edition, consisting of five volumes and featuring contributions from nearly a thousand authors, was published between 2001 and 2012. *Bergey's Manual of Systematic Bacteriology* provided systematic and taxonomic information about individual prokaryotic microbial species, while also delving into aspects of cell organization, physiology, cultivation strategies, genomics, ecology and evolution of particular taxa. In this edition, classification involved determination of the position of a particular microorganism within the phylogenetic trees, while identification of species was based on 16S rRNA gene sequence similarities and phenotypic properties. The change from determinative to systematic bacteriology also represented a continuous improvement in classification criteria, transitioning from differentiations based on morphology and physiology to more comprehensive criteria based on nucleic acid hybridization and the similarity of 16S rRNA gene sequences and later on genome similarities. With the rapid growth of prokaryotic biodiversity exploration, the practice of compiling updated editions in print was unable to keep up with the pace of the primary research. In April 2015, *BMSAB* commenced online publication, which announced the digital era of Bergey's Manual (https://onlinelibrary.wiley.com/doi/book/10.1002/9781118960608) [18] (Table 2). The structure and content of *BMSAB* have continually evolved to reflect advances in our understanding of microbial physiology, genetics, genomics and ecology.

**Table 2.** Evolution of Bergey's Manual.

**Edition**	**Year**	**Name of the manual**	**Editors**	**Type**	**References**
1st	1901	*Determinative Bacteriology*	Frederick DC *et al.*	Print	[70]
1st	1923	*Bergey's Manual of Determinative Bacteriology*	Bergey DH *et al.*	Print	[8]
2nd	1925	*Bergey's Manual of Determinative Bacteriology*	Bergey DH *et al.*	Print	[69]
3rd	1930	*Bergey's Manual of Determinative Bacteriology*	Bergey DH *et al.*	Print	[69]
4th	1934	*Bergey's Manual of Determinative Bacteriology*	Bergey DH *et al.*	Print	[69]
5th	1939	*Bergey's Manual of Determinative Bacteriology*	Bergey DH *et al.*	Print	[82]
6th	1948	*Bergey's Manual of Determinative Bacteriology*	Breed RS *et al.*	Print	[83]
7th	1957	*Bergey's Manual of Determinative Bacteriology*	Breed RS *et al.*	Print	[84]
8th	1974	*Bergey's Manual of Determinative Bacteriology*	Buchanan RE *et al.*	Print	[85]
9th	1994	*Bergey's Manual of Determinative Bacteriology*	Holt JG *et al.*	Print	[81]
1st	2001	*Bergey's Manual of Systematic Bacteriology*	Boone DR *et al.*	Print	[86]
2nd	2012	*Bergey's Manual of Systematic Bacteriology*	Goodfellow M *et al.*	Print	[87]
1st	2015	*Bergey's Manual of Systematics of Archaea and Bacteria*	Whitman WB *et al.*	Digital	[13]

Over the years, Bergey's Manual has embraced the digital age, moving from a printed publication to an online resource. This shift from print to digital underscores the increasing importance of digital technologies in scientific research, providing users with a searchable, up-to-date resource that is accessible globally. The evolution of *BMSAB* reflects the dynamic nature of microbial taxonomy and the ongoing efforts of scientists to better understand the diversity and complexity of prokaryotes. From its humble beginnings as a printed manual to its current status as a comprehensive online reference, *BMSAB* continues to play a critical role in advancing our understanding of the microbial world and providing insights into the natural classification of prokaryotes reflective of their evolutionary history. The value of *BMSAB* varies among the medical, chemical, pharmaceutical and biological sciences, and it especially holds unique significance for the field of microbiology. For students, it supports laboratory courses and serves as an excellent gateway to gain a comprehensive understanding of prokaryotes and their diversity. For researchers in microbiology, medicine and other relevant disciplines, the manual provides authoritative descriptions of various prokaryotic groups. It represents the collective effort and research achievements of nearly a thousand microbiologists worldwide through the Bergey's Manual Trust.

## HISTORICAL OVERVIEW OF THE BERGEY'S INTERNATIONAL SOCIETY FOR MICROBIAL SYSTEMATICS

In the last two decades, significant progress in the isolation and description of novel prokaryotic taxa has occurred. To keep up with the growing interest in the field of prokaryotic systematics and to provide a common platform to disseminate information among microbial taxonomists, Bergey's Manual Trust began discussions on the formation of an international society in 2006–2007. A survey conducted by the Trust along with discussions held at the 2008 International Union of Microbiological Societies meeting in Istanbul in 2008 was highly supportive of the concept. Based on this, the Trust voted to sponsor BISMiS at its annual meeting in 2009. With Prof. James Staley as its founding President, a very generous contribution from Prof. Robert Murray, who also became the first member of the society, and more than 65 other charter members, the society was brought to fruition in 2010. Today, BISMiS has emerged as an international advocate supporting microbial systematics, promoting excellent research in the field and enhancing global communication among taxonomists who study prokaryotes (www.bismis.net).

BISMiS recognizes that the vast diversity of microbial life is one of the largest reservoirs of unknown biological diversity on Earth. Hence, to advance our knowledge about this diversity, microbiologists of many disciplines and countries should be involved. For this, BISMiS provides a common platform for a broad group of microbiologists working across the globe and organizes meetings and discussions to promote research in prokaryotic systematics (Table 3). The inaugural BISMiS meeting took place in Beijing, China in 2011, followed by biennial meetings in Scotland, India and South Africa and returning to China in 2023. The participants of these meetings showcased their engagement in microbial systematics, regardless of their broader research areas within microbiology and their need to understand the changing landscape in this field and its implications for their work. During the meetings, attendees disseminated their most recent discoveries, exchanged ideas and forged enduring friendships that span international and political boundaries. Discussions revolved around shaping the future landscape of prokaryotic systematics, delving into aspects such as the standards and best practices that are integral to the field. At the most recent meeting in November 2023, the dynamic relationship between the ICNP and the SeqCode, coupled with the practical applications, took center stage as a critical roundtable discussion topic. The roundtable discussion provided a platform to explore the relative merits of the two codes of nomenclature and the common goal to best serve the broader microbiology research community, contributing to the ongoing advancements in prokaryotic systematics amidst the challenges presented in the meta-data era. Meetings like this hold enormous value in advancing research in prokaryotic systematics across the globe (Table 3). For example, the last meeting in China has facilitated the expansion of microbial culture collections and contributed to the establishment of genomic data sequencing services, providing researchers with access to valuable data sets [88]. Moreover, the conference has served as a forum for Chinese researchers to engage with their international counterparts and contributed to cooperative advancement of prokaryotic systematics. Over the years, the BISMiS meetings have become a hub for scientists and scholars worldwide, driving significant advancements in prokaryotic systematics.

**Table 3.** BISMiS conferences and major contributions.

**Year**	**Theme**	**Place**	**Sessions**
2011	Microbial Systematics: Concepts, Practices and Recent Advances	Beijing, China	1. Microbial Systematics and Diversity. 2. Technological Advances in Systematics and Biotechnology. 3. Archaeal Systematics and Diversity. 4. Microbial Systematics and Its Impact on Biotechnology.
2014	Defining Microbial Diversity in the Genomic Era	Edinburgh, Scotland	1. Use of Genomic Sequences in Microbial Taxonomy. 2. Chemotaxonomy *in vitro* vs. *in vivo*. 3. Microbial Systematics in the Classroom. 4. Lessons for Systematics from Metagenomic Studies. 5. New Approaches and New Taxa.
2016	Microbial Systematics and Metagenomics	Pune, India	1. Genomic/Metagenomic Description of Novel Taxa. 2. Cultures and Culturing of As-Yet-Uncultivated Microbes. 3. The Role of Cultures in the Twenty-First Century. 4. Modern Approaches to Identification/Diagnosis. 5. Minimum Standards for the Description of New Taxa. 6. Cyanobacterial Taxonomy.
2018	Capturing Species Diversity	Johannesburg, South Africa	1. Updated Taxonomy of Important Taxa. 2. Developments in Taxonomical Practices. 3. Capturing Unique Diversity. 4. Role of Genome Data in Systematics. 5. Bacterial Systematics: What Lies Ahead?
2023	Microbial Systematics in Meta-data Era: Opportunities and Challenges	Guangzhou, China	1. Cultivating Previously Uncultivated Microbes. 2. Changes in Microbial Systematics. 3. How to Name Uncultivated Prokaryotes. 4. Intra- and Inter-Species Diversity and Speciation. 5. Microbial Systematics and Its Impact on Biotechnology Research. 6. Bridging the Gap Between Microbial Systematics and the Larger Microbiology Community.

## CHALLENGES AND OPPORTUNITIES

The current ease and affordability of performing genomic and metagenomic sequencing has generated vast amounts of genomic data, providing valuable insights into the tree of life, transforming prokaryotic systematics from a genome-poor past to a genome- and metagenome-rich era. The key emphasis now lies in optimizing the use of the expanding genomic data, encompassing genomes from both isolates and yet-uncultivated microbes, within the public databases. This ongoing focus remains critical for the advancement of prokaryotic systematics. In the subsequent discussion, we outline four primary challenges confronting prokaryotic systematics and present five strategic propositions discussed at BISMiS aimed at effectively addressing these challenges.

The first challenge involves inferring robust phylogenetic relationships, a critical aspect in prokaryotic systematics. Since 1977, microbial taxonomy has strongly relied on 16S rRNA gene-based phylogeny [89]. However, single-gene phylogenies have many limitations, including low phylogenetic resolution at the highest and lowest taxonomic ranks, incongruity due to multiple copies of 16S rRNA genes in a single organism, missing diversity due to PCR primer mismatches, and PCR-produced chimeric sequences. These limitations corrupted tree topologies and increased the uncertainty of phylogenetic assertions. Although concatenated protein-based phylogenies are considered to be a more accurate approach for constructing phylogenetic trees [90] and are extensively employed in the literature [91–93], it cannot be overlooked that this approach fails to address issues due to horizontal gene transfer events, variations in recombination and differential rates of gene evolution. Furthermore, disparities in the selection of marker genes and tree-building algorithms may lead to differences in the topologies of phylogenetic trees, as it became evident in the distinct outcomes observed for two tree-building methods in the case of candidate phyla radiation (CPR) [93,94]. Therefore, reconstructing phylogenetic trees that accurately reflect the true evolutionary history of the species remains challenging. Nevertheless, the rapid increase in the number of genomes in the last decade has made phylogenomics essential for investigating evolutionary relationships in prokaryotic systematics.

The second challenge arises from the impact of genomes from uncultivated microbes. Currently, the widespread use of metagenomic and single-cell genomic sequencing methods has generated an enormous number of MAGs and SAGs, significantly expanding our understanding of the ‘Tree of Life’ [78]. Simultaneously, the issue of a nomenclature system for uncultivated taxa has been addressed. While the existence of these organisms cannot be denied, the ICNP relegates recommendations on naming them as *Candidatus* taxa to an appendix, which is not part of the legislative code. Although there is currently a proposal to bring *Candidatus* names into the code [95], this proposal deliberately excludes *Candidatus* names from being validly published [27,96,97]. In contrast, the SeqCode allows valid publication of names based on genome sequences, including MAGs and SAGs. There are also differences in reciprocity between the codes. The SeqCode acknowledges validly published names under the ICNP, whereas the ICNP deliberately does not recognize names validly published under the SeqCode as validly published. These conflicts arose because of genuine disagreements within the prokaryotic systematics community; until they are resolved, such disagreement may undermine the common goals of the systematics community, at least temporarily.

The third challenge is how to comprehend the relationships between species, genes and phenotypes. Currently, the functions of a large portion of the genes in microbial genomes remains unknown, and the prediction of the phenotypes from the genotypes is imperfect; particularly, functional studies on the genes of yet-to-be cultured microbial groups are still limited. Accurately predicting phenotypic traits based on genotypic information remains to be advanced by the development of reliable computational tools by utilizing huge amounts of reference data obtained from relevant molecular and biochemical experimentations on cultivated taxa [6,98,99]. One achieved example is the Microbial Trait Analyzer, a tool that could accurately predict various traits related to the microbial utilization of substrates as carbon and energy sources, oxygen requirement, morphology, antibiotic susceptibility, proteolysis and enzymatic activities directly from genome sequences [100].

The fourth challenge is development of rapid identification methods, specifically for clinical needs. With the rapid increase in the number of pure cultures and the parallel discovery of new taxa represented by genomes from yet-uncultivated microbes, developing rapid and standardized species identification schemes remains a challenging task. Despite significant contributions to genome classification by GTDB, the deep-rooted practice of traditional phenotype-based identifications among many medical facilities means that genotypes still need to be mapped to key phenotypes that are used in clinical settings. Furthermore, routine application of chemotaxonomic methods instead of using genomic information to deduce the presence of marker genes may be unnecessary in today's genomic era [101]. Genomic-based analyses are rapidly becoming adopted by clinical microbiology laboratories. However, for the clinical microbiology lab, the confirmation of the expression of particular features is critical for confirming microbiological features that enable effective diagnoses for treating infectious diseases.

With the ever-increasing data and evolving concepts, BISMiS suggests that the future development of prokaryotic systematics and *BMSAB* should focus on the following topics: digitization of information and artificial intelligence (AI), interdisciplinary collaboration, advancement of content, inclusive ideology and community engagement. Given the high number of global genome sequencing efforts underway, the genomes of the type strains of all species will soon be sequenced. The resulting meta-data may influence our perception of currently standardized strategies for deducing novel species identities. BISMiS is committed to ensuring the stable and prosperous development of genomics in the future of prokaryotic systematics.

- **Digitalization of information and AI:** The online publication of *BMSAB* (https://onlinelibrary.wiley.com/doi/book/10.1002/9781118960608) has partially addressed the issue of information updates, enabling the public to follow the most updated taxon descriptions in a single place. Nevertheless, there is need for improvements in the ease of searching and accessing information, both for phenotypes and genome sequences of the prokaryotes, owing to the vast volume of data. This would involve constructing phenotype and genotype databases such as Bacdive (https://bacdive.dsmz.de/) and GTDB (https://gtdb.ecogenomic.org) for all taxonomic ranks or integrating with existing databases, especially for taxa that are associated with human health or other societally important taxa, thereby providing greater convenience for professionals in fields such as medicine and industry. It is crucial to promote data sharing and interoperability with global resources to enhance the quality of descriptions provided in *BMSAB*. This includes initiatives such as the establishment of global or national data centers that provide more efficient and increasingly available services for microbial genome sequencing and data storage and dissemination, which will contribute to the greater microbiology research enterprise. Additionally, although the approach for prokaryotic systematics relying on relative evolutionary divergence (RED) represents a significant innovation, there is still a lack of theoretical breakthroughs. The current understanding of systematics continues to be heavily reliant on biomarker genes and tree-building methods. The introduction of artificial intelligence will assist in deciphering data and advancing prokaryotic-systematics-related fields, such as deep learning and machine-learning-based taxonomy analyses and prokaryotic identification methods that abridge both complex experimental and analytical procedures [102–104].
- **Interdisciplinary collaboration:** Prokaryotic systematics encompasses the study of phenotypes and genotypes of taxa. In recent years, researchers have shown that the integration of microbiology, biochemistry, physics and computer science can play a crucial role in improving the classification, identification and description of prokaryotes. Additionally, leveraging genomics, chemistry (e.g. protein-based identification) [51,105,106], physics (e.g. Raman-spectroscopy-based identification), multi-omics-based identification and machine-learning-based prediction and identification of microbial taxa can enhance the reliability of microbial classification and identification and enable high-throughput analysis.
- **Advancement of content:**  *BMSAB* currently emphasizes the classification of microorganisms that are available as pure cultures, and has limited descriptions of microbial groups that are not available as pure cultures. A significant number of yet-uncultivated microorganisms exist in the environment, sometimes referred to as ‘microbial dark matter’ [107,108]. In the future, as a greater number of organisms are described based on cultivation-independent studies, *BMSAB* will endeavor to include them in future chapters, whether they are described under ICNP as *Candidatus* or validly published under the SeqCode. Furthermore, a larger effort is needed by the scientific community who make use of this information to help expand it and keep it updated.
- **Inclusive and collaborative ideology:** Differences in perspectives exist between traditional and modern taxonomists, and many members of the scientific community have arrived at a consensus in the face of such disparities [25]. It is worth noting that the existence of two separate codes of nomenclature dedicated to prokaryotes is a challenge that needs to be addressed to harmonize and simplify prokaryotic systematics for the greater microbiology research community. While the current situation may result in some degree of confusion due to two parallel codes of nomenclature, it should be stressed that taxonomic analyses and assigning names to prokaryotes should maintain scientific standards, such as the necessity for being able to reproduce data. However, it is also recognized that the collection and archiving of genome sequence data of the as-yet uncultivated prokaryotes is necessary for, perhaps, some of the most interesting and exciting microbiological studies that are being carried out today. It is the dedication of expert scholars, driven by the motivation for the subject's prosperity, that will overcome these challenges. This spirit of inclusivity is crucial for the future of prokaryotic systematics, and BISMiS is committed to fostering this belief.
- **Community engagement:** The active participation and involvement of the scientific community are important for any branch of science to flourish. With this in mind, BISMiS Live was initiated in 2021 as a monthly online seminar series to engage researchers who share a passion for prokaryotic systematics from across the globe (https://bismis.net/bismislive.html). So far, more than 4200 participants have benefited from 33 sessions on this platform, which included scientific presentations from experts, lively debates and discussion sessions spanning different research areas, and current hot topics within the field. A major goal of BISMiS Live is to promote frequent scholarly exchanges by delivering fresh perspectives on important topics in systematics. Fostering the interests of young scholars is essential for the vitality of microbiology and taxonomy, and BISMiS Live strives to achieve this.

## CONCLUDING REMARKS

This review retrospectively examines the history of prokaryotic systematics and the century-long contributions of *BMSAB* to this field along with its much younger but impactful partner BISMiS. It aims to elucidate the current state of development and highlights the transformative shift from ‘polyphasic taxonomy’ to the ‘meta-data era’. This evolution mirrors the progressing comprehension of development within the microbiological community. As a venerable witness to the development of prokaryotic systematics, *BMSAB* encapsulates the unwavering dedication and persistent efforts of generations of expert scholars. In the contemporary landscape of genomics and bioinformatics, the manual is poised to more clearly reflect the strides made in prokaryotic systematics, ensuring its relevance, currency and innovative character. BISMiS, in collaboration with the ICNP and the SeqCode, is committed to advancing scientific openness, fostering cooperative mechanisms and amplifying the pivotal role of prokaryotic systematics in the field of microbiology. This collective endeavor will help to illuminate a path towards a brighter human future, in which we will have a complete systematic description of the prokaryotic world.
